# Study Design of the Global Prospective Hemolytic Disease of the Fetus and Newborn Registry (GERANIUM)

**DOI:** 10.1055/a-2816-3361

**Published:** 2026-03-20

**Authors:** May Lee Tjoa, Ashley Orillion, Raymond Mankoski, Nida Imran, Carol Mao, Alexis Krumme, Sylvie Van Hoorde, Blanca Linares-Rivas Rico, Yosuke Komatsu

**Affiliations:** 1 Johnson & Johnson, Cambridge, Massachusetts, United States; 2 Johnson & Johnson, Horsham, Pennsylvania, United States; 3 The NemetzGroup, Boston, Massachusetts, United States; 4 Johnson & Johnson, Beerse, Belgium; 5 Johnson & Johnson, Madrid, Spain; 6 Johnson & Johnson, Spring House, Pennsylvania, United States

**Keywords:** fetal hydrops, hemolytic disease of the fetus and newborn, intrauterine transfusion, IUT, intravenous immunoglobulin, IVIg, registry, study design

## Abstract

**Objective:**

Hemolytic disease of the fetus and newborn (HDFN) is a rare and serious immune-mediated condition that can result in poor fetal and neonatal outcomes. During pregnancy, monitoring with middle cerebral artery Doppler ultrasound and intrauterine transfusions (IUTs) are the current standard of care, although intravenous immunoglobulin (IVIg) has been adopted in some centers to attempt to delay the onset of fetal anemia. Significant gaps remain in understanding the safety and effectiveness of current HDFN management, both during pregnancy and in neonates and children. We present the design of the Global Prospective Hemolytic Disease of the Fetus and Newborn Registry (GERANIUM; ClinicalTrials.gov Identifier: NCT07194070), a study that will provide real-world, prospective, longitudinal data on management, clinical course, and outcomes of HDFN from the antenatal period up to 2 years after birth.

**Study Design:**

GERANIUM is a prospective, observational study enrolling alloimmunized pregnant individuals (aged ≥18 years) before 24 weeks of gestational age who are at high risk for HDFN based on previous obstetric history. This study will enroll approximately 175 participants across multiple global sites. Data collected during pregnancy include fetal loss, frequency and severity of HDFN, treatment patterns, as well as effectiveness and adverse outcomes of IUTs and other interventions. Following birth, infants will be followed for the first 2 years of life, with documentation of HDFN sequelae, including treatments, growth, and development. Impact on the parents and children will be assessed using validated participant- and observer-reported outcome measures.

**Conclusion:**

The GERANIUM registry will provide prospective, contemporaneous data on current management of HDFN and associated outcomes.

**Key Points:**

- HDFN can result in fetal loss or poor long-term outcomes.
- IUT and IVIg can be associated with serious complications.
- Impacts of HDFN on patients' quality of life are unclear.
- GERANIUM will provide contemporaneous data on HDFN care.
- Prospective data for mothers/infants will be collected.

## Introduction

Hemolytic disease of the fetus and newborn (HDFN) is a rare and potentially life-threatening immune-mediated condition caused by maternal alloantibody production due to incompatibility between maternal and fetal blood group antigens. 1 2 3 4 These alloantibodies cross the placenta and bind to fetal red blood cells (RBCs), leading to hemolysis and anemia. 4 5 Rhesus D antigen (Rh(D)) alloimmunization occurs in an estimated 1 to 3 per 1,000 Rh(D)-negative pregnancies despite the availability of Rh(D) immunoprophylaxis. 6 7 Rh(D) incompatibility is the most common cause of severe HDFN, accounting for 64.1% of all HDFN cases. 8 Severe HDFN is rare, and a prior history of severe disease greatly increases recurrence risk in subsequent pregnancies, often with earlier onset due to repeated antigen exposure. 9 10 11 12 Severe disease can lead to fetal hydrops, preterm birth, and, after delivery, complications such as hyperbilirubinemia, kernicterus, and persistent anemia requiring plasma exchange or transfusions. 3 4 13 14 15 16 17 18 If severe fetal or neonatal anemia is not adequately managed, there is a high risk of infant mortality. 3 19

Current standard of care (SoC) in the antenatal setting includes monitoring for fetal anemia using middle cerebral artery (MCA) Doppler ultrasound. If severe anemia is confirmed by cordocentesis in alloimmunized pregnant individuals, intrauterine transfusions (IUTs) of RBCs are performed. 20 21 However, IUT is a surgical intervention associated with both procedural risks and short-/long-term risks to the mother and fetus, including emergency cesarean delivery, premature birth, fetal loss, intrauterine infections, and increased maternal alloimmunization. 14 16 22 23 24 In some centers, intravenous immunoglobulin (IVIg) and plasma exchange are used to delay the onset of anemia and the need for an IUT. 25 26 27 However, prospective data to support the effectiveness of IVIg in this setting are limited, and the need for subsequent IUTs remains. 25 26 28 29 Although diagnostics for identifying Rh(D) alloimmunization have improved over time, many patients require intervention with these prenatal treatments; results from a systematic literature review suggested that IUTs were performed in 13% of pregnancies with a positive anti-Rh(D) screen, and IUT combined with IVIg was administered in 3.2% of Rh(D) alloimmunization cases and 4.3% of Kell alloimmunization cases. 1

The large population-based studies conducted to date have been retrospective analyses. 14 15 18 While these studies have provided valuable insights, significant gaps remain in the availability of data on current HDFN management and outcomes. Most existing evidence reflects experience from specialized settings in limited geographies, which may not be representative of broader clinical practice. In addition, SoC and therapeutic options for HDFN have evolved in recent years, making it essential to capture contemporary, real-world data across diverse settings. Here, we report the design of the Global Prospective Hemolytic Disease of the Fetus and Newborn Registry (GERANIUM), which aims to provide prospective, longitudinal, contemporaneous data on current HDFN interventions and outcomes in a real-world cohort of pregnant individuals and their corresponding fetuses, neonates, infants, and children. GERANIUM will also provide data regarding longer-term outcomes on child development, health-related quality of life (HRQoL), medical resource utilization due to HDFN, and biomarkers. The data derived from GERANIUM may provide evidence to increase scientific knowledge, support the development of evidence-based guidelines, provide context for emerging therapies for HDFN, and inform future research directions.

## Materials and Methods

### Ethics

GERANIUM (ClinicalTrials.gov Identifier: NCT07194070) is currently being conducted in compliance with a prespecified protocol and analysis plan in conformance with applicable regulatory requirements. All participating centers will obtain approval from their respective institutional review boards or independent ethics committees prior to the start of the study at their site. All participants will provide written informed consent before any data collection. Each site will maintain documentation of informed consent in accordance with its local regulations and applicable regulatory requirements.

### Participants

Pregnant individuals aged ≥18 years, with an estimated gestational age of up to 24 weeks, and with evidence of alloimmunization in the current pregnancy, along with an antigen-positive fetus corresponding to the current maternal alloantibody, may be enrolled ( Table 1 ). Eligible participants are required to have a history of alloimmunization in a previous pregnancy that resulted in HDFN, demonstrated by ≥1 of the following: fetal anemia diagnosed by MCA Doppler ultrasound, received ≥1 IUT, fetal hydrops, stillbirth with fetal or placental pathology indicative of HDFN, neonatal exchange transfusion, neonatal simple transfusion, neonatal hyperbilirubinemia, or positive direct antiglobulin test in the neonate. Individuals who are participating in an interventional trial of an investigational agent or who are at risk for HDFN due to ABO incompatibility alone are excluded.

**Table 1:** Eligibility criteria

**Inclusion criteria**
• Pregnant individuals aged ≥18 years • Pregnant with an estimated gestational age of up to 24 weeks based on ultrasound dating • History of a previous alloimmunized pregnancy that included ≥1 of the following: ▪ Fetal anemia diagnosed by MCA Doppler ultrasound ▪ Received ≥1 IUT as a result of HDFN ▪ Fetal hydrops ▪ Stillbirth or fetal demise with fetal or placental pathology indicative of HDFN ▪ Neonatal exchange transfusion due to HDFN ▪ Neonatal simple transfusion due to HDFN ▪ Neonatal hyperbilirubinemia due to HDFN ▪ Positive DAT in neonate • Documented presence of maternal alloantibody based on local laboratory results during current pregnancy • Evidence of an antigen-positive fetus corresponding to the current maternal alloantibody: ▪ Fetal antigen status confirmed by cffDNA, or ▪ Fetal antigen status confirmed by amniocentesis, or ▪ Paternal genotype confirmed • The pregnant participant or legally acceptable representative must provide informed consent (per local regulations or ethics committee requirements) for the collection and use of their medical data and the medical data for their corresponding fetuses/neonates/infants/children • The pregnant participant agrees to sign the Release of Medical Information Form or equivalent document, according to local regulation, by all persons who are required to give consent, thereby permitting the participant's health care provider(s) and pediatric health care providers to be contacted for medical information
**Exclusion criteria**
• Active participation in an interventional trial of an investigational agent • At risk for HDFN due to ABO being the sole alloimmunization antigen in the current pregnancy (i.e., ABO plus another antigen is permissible)

Abbreviations: cffDNA, cell-free fetal deoxyribonucleic acid; DAT, direct antiglobulin test; HDFN, hemolytic disease of the fetus and newborn; IUT, intrauterine transfusion; MCA, middle cerebral artery.

### Study Design

**Fig. 1:**
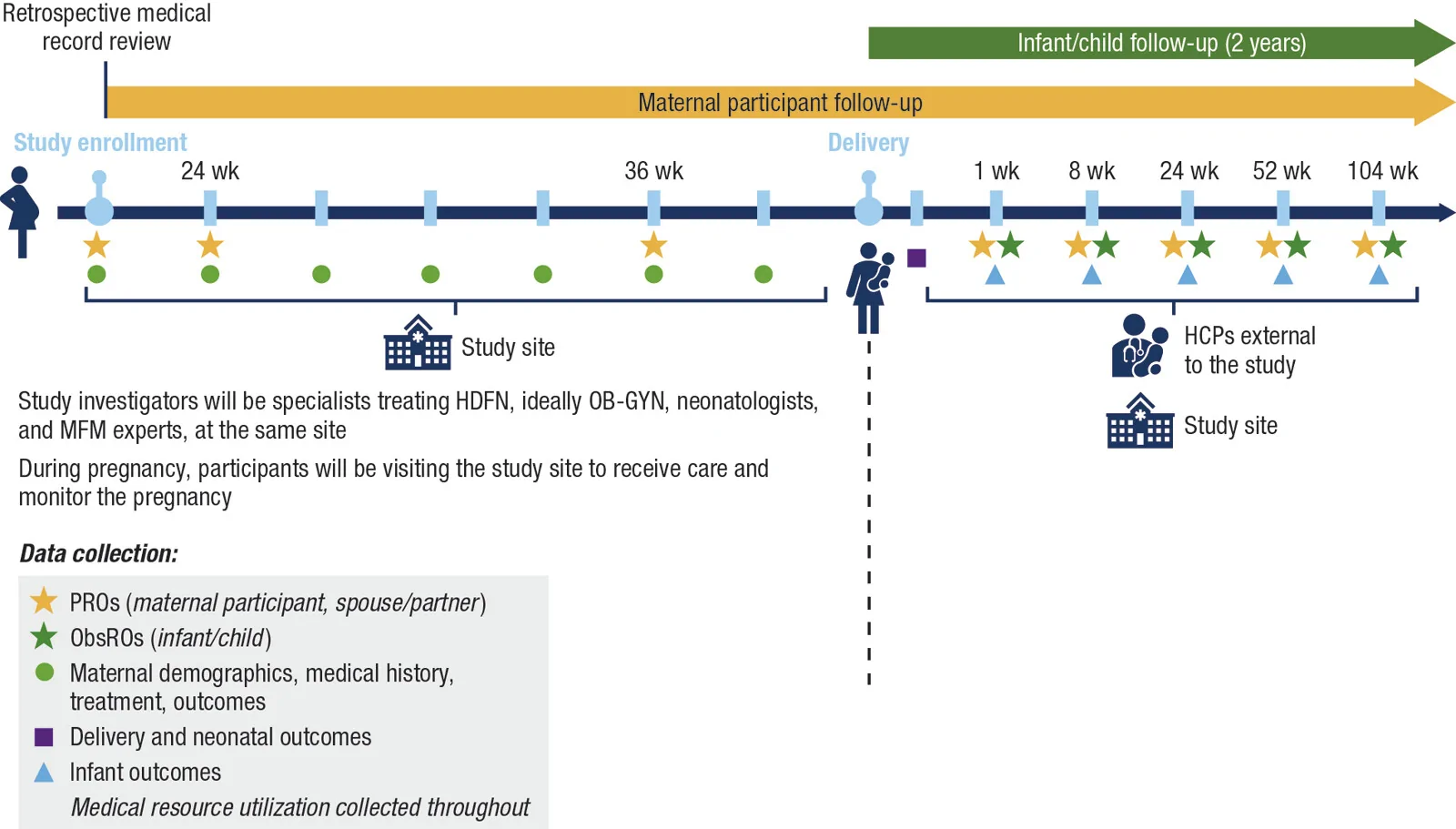
GERANIUM registry study design. HCP, health care professional; HDFN, hemolytic disease of the fetus and newborn; MFM, maternal–fetal medicine; OB-GYN, obstetrician-gynecologist; ObsRO, observer-reported outcome; PRO, patient-reported outcome.

GERANIUM is a global, prospective, observational, longitudinal, non-interventional registry of pregnancies affected by HDFN. Data will be collected from eligible pregnant participants during pregnancy from enrollment (on or before 24 weeks of gestational age) and for 2 years after delivery, and from their infants for 2 years after birth ( Fig. 1 ). Participants will be recruited from medical centers that routinely diagnose and manage pregnant individuals at risk for HDFN over a period of approximately 5 years. All treatment and clinical management will be conducted following local practice and regulations, as determined by the principal investigator or treating physician. No treatment will be provided by the sponsor as part of this study, and participants will not be discontinued from the registry if they receive any specific interventions, except for an investigational agent received as part of a clinical trial. No study visits, clinical assessments, or procedures will be required.

### Data Collection

At enrollment, data will be collected from the medical records or participant reports. These data will include demographic characteristics, medical and obstetric history, history of HDFN, diagnosis of HDFN, non-HDFN pregnancy complications, comorbidities, treatments, procedures, adverse outcomes, and fetal assessments.

Antenatal data will be collected retrospectively at enrollment and prospectively after enrollment and will include diagnosis of HDFN, date of HDFN diagnosis, non-HDFN–related pregnancy complications, fetal assessments, HDFN treatment use and patterns, and treatment-related adverse outcomes. HDFN treatments include IUT, IVIg, and plasma exchange as a result of HDFN. Biomarker data, including alloantibody titers and cell-free fetal DNA, may be recorded during routine blood draws. If available, umbilical and uterine artery flow measurements using MCA Doppler will be recorded. In the case of a multifetal pregnancy, data will be collected for each fetus. All clinical assessments will be at the discretion of the physician, depending on the presence and severity of HDFN symptoms.

Pregnant participants will be asked to complete patient-reported outcome (PRO) and observer-reported outcome (ObsRO) assessments to evaluate HRQoL, anxiety and stress, the impact of HDFN on adult family members or partners, and infant/child well-being ( Fig. 1 ). All PRO and ObsRO assessments will be voluntary. Medical resource utilization will be documented, including hospitalizations, medical care encounters (emergency room, outpatient, and inpatient), diagnostic tests and procedures, and medications.

At birth, data collected will include gestational age at delivery, mode of delivery, placental evaluation, birth weight, sex, Apgar score at 1 and 5 minutes, neonatal laboratory evaluations at birth, neurodevelopmental assessments, and perinatal complications (including preterm premature rupture of membranes, birth asphyxia, hypoxic–ischemic encephalopathy, and fetal death).

During the postnatal period, information will be collected regarding infant health care resource utilization (e.g., hospitalizations, length of hospital stay, length of intensive care unit stay, rates of emergency department outpatient visits, urgent care and outpatient visits for HDFN parental participants), adverse outcomes related to SoC or concomitant medications, clinical laboratory evaluations (e.g., bilirubin level, platelet count, leukopenia, thrombocytopenia, maximum ferritin), treatments (e.g., phototherapy, IVIg, exchange and simple transfusions, erythropoietin treatment), outcomes (e.g., hyperbilirubinemia, necrotizing enterocolitis, respiratory distress syndrome, sepsis, cerebral injury, kernicterus, major birth defects, assisted feeding type, mortality). Growth and neurodevelopmental outcomes will be collected up to 2 years of life among all liveborn infants, with parent or caregiver permission required for formal enrollment and neurodevelopmental assessments of their child in the study. The modified Neonatal Morbidity and Mortality Index (mNMMI) will be assessed to categorize the severity of outcomes. 30 After hospital discharge, growth and development (e.g., height, weight, head circumference) and neurodevelopmental assessments (Bayley scales) will be collected for up to 2 years. During this period, comorbid conditions will be documented, and health care resource utilization outcomes will be documented.

### Study Endpoint

A listing of study endpoints is provided in Table 2 . The primary endpoint of the study is the proportion of pregnancies in participants at risk for HDFN receiving SoC that do not result in fetal loss (due to any reason), IUT, fetal hydrops, or neonatal death (due to any reason) during the neonatal period. Key secondary endpoints include the severity of HDFN, measured using a composite severity index, timing of first IUT or fetal hydrops, number of IUTs received, and risk of mortality and morbidity in neonates born to participants at risk for HDFN using the mNMMI.

**Table 2:** Study objectives and endpoints

Primary objective	Primary endpoint
To evaluate the risk of anemia in fetuses and neonates of pregnant participants who are at risk for HDFN and receiving SoC	Proportion of pregnancies that do not result in fetal loss (due to any reason), IUT, fetal hydrops, or neonatal death (due to any reason) through the neonatal period
Secondary objectives	Key secondary endpoints
To describe the severity of HDFN as measured by a composite severity index in pregnant participants and their neonates/infants at risk for HDFN and who are receiving SoC	The severity of HDFN is measured by a composite HDFN severity index and is defined as: • 5 (fatal): Fetal or neonatal death due to any reason • 4 (severe): Fetal hydrops (in fetus or newborn) or receiving IUT during pregnancy as a result of HDFN, but not 5 (fatal) • 3 (moderate): Neonatal exchange transfusions received as a result of HDFN-related hemolysis and jaundice, but not 4 (severe) or 5 (fatal) • 2 (mild): Neonatal simple transfusions received due to HDFN after birth, with or without phototherapy, but not 3 (moderate), 4 (severe), or 5 (fatal) • 1 (minimal or none): Not 2 (mild), 3 (moderate), 4 (severe), or 5 (fatal) as described above. HDFN requiring phototherapy will be classified in this category
To describe the timing of the onset of HDFN in pregnant participants at risk for HDFN who are receiving SoC	Time to first occurrence of IUT or fetal hydrops
To describe the risk of mortality and morbidity in neonates born to participants at risk for HDFN who are receiving SoC	mNMMI in liveborn neonates
To describe antenatal management and outcomes in pregnant participants at risk for HDFN who are receiving SoC	Key secondary endpoint: • Number of IUTs received during pregnancy Other endpoints: • Proportion of pregnancies with fetal loss • Proportion of pregnancies with fetal or neonatal death (through the neonatal period) as a result of HDFN • Proportion of pregnancies with fetal hydrops • Proportion of pregnancies receiving IUT during pregnancy • GA at first IUT • Proportion of pregnancies receiving >1 IUT during pregnancy • The proportion of pregnancies receiving IUT or HDFN resulting in fetal demise before GA week 20 ^0/7^ • GA at delivery • Number of IVIg treatments received and dosage, among pregnancies with ≥1 IVIg, GA at first dose administration, and frequency of administration • Number of plasma exchange treatments received and dosage, among pregnancies with ≥1 plasma exchange treatment, GA at first dose administration, and frequency of administration • Any additional treatment for HDFN
Other objectives	Other endpoints
To describe the neonatal management and other outcomes in neonates/infants of pregnant participants at risk for HDFN who are receiving SoC	• Proportion of pregnancies with neonatal death through the neonatal period • Proportion of liveborn neonates with HDFN-related morbidities other than anemia and hyperbilirubinemia/jaundice • Absolute weight and weight changes over time from birth through week 104 in liveborn neonates/infants • Liveborn neonates' length of stay in the neonatal intensive care unit • Proportion of liveborn neonates receiving exchange transfusions for HDFN • Number of neonatal exchange transfusions per liveborn neonate • Proportion of liveborn neonates/infants with simple transfusions for HDFN up to 12 weeks after birth • Number of simple transfusions for HDFN per liveborn neonate/infant up to 12 weeks after birth • Proportion of liveborn neonates with hyperbilirubinemia treated with phototherapy • Number of days of phototherapy received for hyperbilirubinemia per liveborn neonate • Proportion of liveborn neonates/infants receiving IVIg for HDFN treatment
To describe the adverse outcomes of treatment in pregnant participants at risk for severe HDFN who are receiving SoC	Maternal–fetal adverse outcomes: • Maternal death, adverse outcomes related to SoC treatment, infections, serious infections, infusion reactions, hypersensitivity reactions • Maternal pregnancy complications • IUT-related complications Pregnancy outcomes: • Proportion of pregnancies with cesarean delivery, cesarean delivery due to IUT complications, preterm birth (GA weeks <28 ^0/7^ , <32 ^0/7^ , <34 ^0/7^ , and <37 ^0/7^ ), fetal growth restriction, and preeclampsia
To describe the adverse outcomes of treatment in neonates/infants born to SoC-treated pregnant participants	Neonate/infant adverse outcomes and development: • Proportion of liveborn neonates/infants who died • Adverse outcomes related to SoC, infections, and serious infections • Proportion of liveborn neonates/infants receiving IVIg for non-HDFN indications • Proportion of liveborn neonates/infants with abnormal hearing • Bayley Scales of Infant and Toddler Development at weeks 52 and 104 in infants • Treatment-related neonatal outcomes (adverse outcomes related to treatment), including severity and causality • Non–treatment-related neonatal outcomes (adverse outcomes not related to treatment), including severity and causality
To describe participant, caregiver, and spouse-/partner-reported outcomes in participants at risk for HDFN who received SoC	Parental participant PROs at each time point: • SF-36 v2 scores for acute domains and component scores • EQ-5D-5L VAS and EQ-5D-5L index scores • GAD-7 scores • PSS-10 scores ObsROs for infant/child: • Summary of ITQOL-SF47 questionnaire score during the study • Summary of IQI score over time Spouse-/partner-reported outcome: • Change from baseline of FROM-16 score over time
Exploratory objectives	Exploratory endpoints
To evaluate longer-term outcomes (clinical, economic, and humanistic) in the children of participants	• Baseline and change from baseline in neonatal/infant/child measures of growth and development (e.g., length/height, weight, BMI, and head circumference) relative to age- and sex-matched regional norms • Proportion of neonates/infants/children with abnormal growth, development, and hearing • Proportion of neonates/infants/children vaccinated • Proportion of children with cerebral palsy, motor/sensory disorders, other neurodevelopmental disorders or behavioral disorders, and dental disorders
To describe the longer-term severity of HDFN as measured by a composite severity index in pregnant participants and their neonates/infants at risk for HDFN and receiving SoC	The severity of HDFN as measured by a composite HDFN severity index and defined as: • 5 (fatal): Fetal or neonatal death due to any reason • 4 (severe): Hydrops fetalis (in fetus or newborn) or receiving IUT during pregnancy as a result of HDFN, but not 5 (fatal) • 3 (moderate): Neonatal exchange transfusions received as a result of HDFN-related hemolysis and jaundice, but not 4 (severe) or 5 (fatal) • 2 (mild): Neonatal simple transfusions received due to HDFN after birth, with or without phototherapy, but not 3 (moderate), 4 (severe), or 5 (fatal) • 1 (minimal or none): Not 2 (mild), 3 (moderate), 4 (severe), or 5 (fatal) as described above. HDFN requiring phototherapy will be classified in this category ▪ Note: Data through 12 weeks after birth will be included
To describe the treatment pathway and medical resource utilization associated with an HDFN diagnosis and with associated treatment modalities	• Rates of resource utilization outcomes (e.g., hospitalization, length of hospital stay, length of ICU stay, and rates of ER/urgent care visits, same-day visits for treatments/procedures in an outpatient facility, and outpatient visits with health care professionals for pregnant participants and their corresponding neonates/infants/children) • Parental referral of pregnant participant and neonate/child to treatment centers and information about the path to referral (e.g., diagnosing physician, treating physician, referral center, academic medical center)
To assess risk factors for the development of HDFN and responsiveness to treatment	Relevant biomarker and clinical data for testing and creating a risk-stratification model and evaluating diagnostic tests, including: • Clinical characteristics (e.g., obstetric history, GA, prior IUT) • Fetal hemoglobin, hematocrit, and alloantibody levels at first IUT and in subsequent IUTs • Pregnant participant serum levels of IgG1, IgG2, IgG3, IgG4, IgA, IgM, and IgE • Slope of MCA-PSV by Doppler ultrasound

Abbreviations: BMI, body mass index; EQ-5D-5L, EuroQol 5-Dimension 5-Level Questionnaire; ER, emergency room; FROM-16, Family Reported Outcome Measure; GA, gestational age; GAD-7, Generalized Anxiety Disorder 7-item; HDFN, hemolytic disease of the fetus and newborn; ICU, intensive care unit; Ig, immunoglobulin; IQI, Infant health-related Quality of Life Instrument; ITQOL-SF47, Infant/Toddler Quality of Life Short Form-47 Item; IUT, intrauterine transfusion; IVIg, intravenous immunoglobulin; MCA-PSV, middle cerebral artery peak systolic velocity; mNMMI, modified Neonatal Morbidity and Mortality Index; ObsRO, observer-reported outcome; PRO, patient-reported outcome; PSS-10, Perceived Stress Scale 10 item; SF-36 v2, Short Form 36 Health Survey version 2; SoC, standard of care; VAS, visual analog scale.

### Data Analyses

The registry aims to enroll approximately 175 pregnant participants across approximately 21 planned study sites in North America, Latin America, Europe, and Asia Pacific. This sample size is based on achievable estimates given enrollment into past and ongoing interventional HDFN studies. 30 The number of neonates/infants/children is based on the assumptions related to live birth percentages and the potential loss to follow-up. 31 A sample size requirement has not been defined, as there is no formal hypothesis testing planned, and the results will be presented descriptively. As this registry is based on real-world clinical practice, it is anticipated that data may be missing due to tests not having been conducted or due to a lack of data availability. As not all maternal participants are expected to remain enrolled after birth, some attrition in infant follow-up data is anticipated. Additionally, there are no formal safety analyses for this observational registry study describing treatments received and outcomes in an SoC setting. Data from the registry will be disseminated at regular intervals during the study.

## Discussion

The prospective, global, non-interventional GERANIUM registry will address the paucity of current data on HDFN by systematically documenting current treatment patterns and effectiveness, as well as maternal, fetal, neonatal, infant, and child outcomes from early pregnancy through 2 years of life. In addition to clinical measures, the study will capture the broader impact of HDFN on pregnant participants, the well-being of their children, and their families, an area where evidence is particularly limited. 32 The linkage between mother and baby will enable the assessment of longer-term outcomes in the context of obstetric history, offering a more complete picture of disease treatments, participant and family unit impact, and health care impacts. Taken together, these efforts will provide evidence to advance scientific knowledge, support the development of evidence-based guidelines, and support hypotheses for future research.

A key strength of this registry is its prospective design, which contrasts with the largely retrospective nature of prior studies. Additionally, the unique inclusion of PROs and ObsROs for pregnant participants, which have not been well-captured within the current SoC, is a currently unavailable resource that will enable enhanced interpretation of the impact of HDFN. 14 15 18 Certain limitations associated with this study should be acknowledged. Eligible pregnant individuals may choose not to participate, potentially introducing selection bias; for instance, those who are more severely affected before 24 weeks of gestation might be less likely to enroll, and those with severely affected children might be more likely to discontinue participation. Additionally, as enrollment in the registry is restricted to pregnant participants with a previous pregnancy affected by HDFN, the study cohort may represent more severe cases than observed in the general population of alloimmunized pregnancies, and nulliparous pregnancies will not be captured. Missing data may occur due to the types of assessments, especially infant-related data from health care providers, or loss to follow-up.

## Conclusion

The GERANIUM registry is a global, prospective, longitudinal, non-interventional registry of pregnancies affected by HDFN. This registry is designed to evaluate current HDFN interventions, as well as long-term infant outcomes, HRQoL, medical resource utilization, and select optional biomarkers associated with HDFN risk, in a real-world setting. Data from GERANIUM will provide a unique contemporary insight into HDFN and its management globally.
